# Are obesity risk genes associated with binge eating in adolescence?

**DOI:** 10.1002/oby.21147

**Published:** 2015-07-20

**Authors:** Nadia Micali, Alison E Field, Janet L Treasure, David M Evans

**Affiliations:** 1 Population, Policy and Practice Research Programme, Child and Adolescent Mental Health Palliative Care and Pediatrics Section, Institute of Child Health, University College LondonLondon, UK; 2 Division of Adolescent Medicine, Department of Medicine, Boston Children's Hospital and Harvard Medical SchoolBoston, Massachusetts, USA; 3 Channing Division of Network Medicine, Department of Medicine, Brigham and Women's Hospital and Harvard Medical SchoolBoston, Massachusetts, USA; 4 Department of Epidemiology, Harvard T.H. Chan School of Public HealthBoston, Massachusetts, USA; 5 Section of Eating Disorders, Psychological Medicine, King's College London, Institute of PsychiatryLondon, UK; 6 MRC Integrative Epidemiology Unit, University of BristolUK; 7 School of Social & Community Medicine, University of BristolBristol, UK; 8 University of Queensland Diamantina Institute, Translational Research InstituteBrisbane, Queensland, Australia

## Abstract

**Objective:**

Cognitions and behaviors characteristic of binge eating are associated with a polymorphism in the *FTO* gene, robustly related to body mass index (BMI) and obesity risk. We investigated the association between binge eating and the individual and combined effect of 32 SNPs robustly associated with BMI in a population-based sample. We hypothesized that higher BMI and binge eating might share a common genetic etiology.

**Methods:**

Binge eating was assessed in adolescents from the Avon Longitudinal Study of Parents and Children at age 14 (*n* = 5,958) and 16 years (*n* = 4,948). We tested associations between 32 BMI-related SNPs and binge eating in crude and BMI-, age-, and gender-adjusted regression models.

**Results:**

Crude analyses showed an association between binge eating and rs1558902 (*FTO*) that persisted after adjustment for BMI (OR = 1.20, *P* = 8 × 10^−3^). A weighted allelic score consisting of all 32 BMI-related SNPs was associated with binge eating (*P* = 8 × 10^−4^); this association attenuated (*P* = 0.08) when rs1558902 was removed from the weighted allelic score.

**Conclusions:**

BMI-related genes are associated with adolescent binge eating, in particular an *FTO* polymorphism. Although replication is needed, our findings have biological plausibility and are consistent with a postulated effect of *FTO* on appetite and food intake. Future studies should aim to understand the mechanisms underlying the relationship between *FTO*, binge eating, and obesity.

## Introduction

Binge-eating disorder (BED) is the most common eating disorder (ED) in the general population, with a lifetime prevalence of about 1.5% in adolescents and adults 1,2, and is associated with adverse physical and psychological outcomes 3,4. BED is characterized by episodes of overeating with loss of control (occurring on average once a week over 3 months) and accompanied by distress. BED has recently been recognized as a diagnostic category in DSM5 5. The prevalence of engaging in binge eating behaviors, both in the context of a full-criteria ED diagnosis or in the absence of other ED features, is about 10% in adults 6 and adolescents 7. Binge eating is most common in individuals who are overweight/obese 8.

It is widely accepted that ED are partly explained by genetic factors 9. However, since BED was only recently recognized as an official disorder, few studies have investigated genetic risk for BED. Despite the paucity of research there is initial evidence that genetic factors influence risk for BED 9. Heritability estimates range between 0.39 10 in a mixed-gender sample of Norwegian twins and 0.45 in a female-only twin sample 11. Evidence from twin studies shows a moderate correlation between obesity and binge eating, which suggests that some of the same genetic factors might influence both obesity and binge eating 12.

Genetic association studies have investigated risk-conferring genes particularly in the dopamine, serotonin, and appetitive systems. Two studies found an association between *MC4R* (melanocortin 4 receptor) variants and BED 13,14, but another study failed to find an association 15. A more recent study of 289 youth aged between 6 and 19 years found an association between a polymorphism in the *FTO* locus and loss-of-control eating, which is one of the main characteristics of BED/binge eating behavior 16. Similarly a recent study showed an association between a weighted allelic risk score, obtained from combining the effect of 32 SNPs robustly associated with body mass index (BMI), and emotional and uncontrolled eating 17, behaviors highly correlated with binge eating 18.

Based on these studies and evidence from our studies that BED predicts overweight 3, we aimed to: 1 investigate whether variants previously identified as associated with BMI 17 were also associated with adolescent binge eating (overeating with loss of control) and 2 test the role of a polygenic weighted allelic score [obtained by combining the 32 independent genetic variants associated with BMI from a genome-wide association study 19]. We also aimed to explore whether associations varied by gender and frequency of binge eating at each time-point.

## Methods

### Participants

The Avon Longitudinal Study of Parents and Children (ALSPAC) is a longitudinal, population-based, prospective study of women and their children 20. All pregnant women living in the geographical area of Avon, UK, who were expected to deliver their baby between 1st April 1991 and 31^st^ December 1992 were invited to take part in the study. All women gave informed and written consent. The study website contains details of all the data that are available through a fully searchable data dictionary (http://www.bris.ac.uk/alspac/researchers/data-access/data-dictionary).

The children from 14,541 pregnancies were enrolled; 13,988 children were alive at 1 year. At age 14 years 10,303 adolescents (singletons) were eligible for follow-up 20 and were sent questionnaires; 5,958 adolescents (57.8% of those eligible) returned completed questionnaires. At age 16 years 9,660 adolescents (singletons) were sent questionnaires and 4,948 returned them (51.2%).

Ethical approval for the study was obtained from the ALSPAC Ethics and Law Committee and the Local Research Ethics Committees.

### Binge eating

Binge eating was assessed using a two-part question (21). Participants were first asked about the frequency during the past year of eating a very large amount of food. Those who answered yes were directed to a follow-up question that asked whether they felt out of control during these episodes, i.e., whether they could not stop eating even if they wanted to stop. Adolescents who answered yes to both questions were classified as engaging in binge eating.

For the purpose of the study we used binge eating either at 14 or 16 years of age as an outcome. We also created an ordinal variable for frequency of bingeing reported in the year prior to assessment (never, < once a month, one to three times per month, once a week, >once a week), in order to determine whether increasing frequency of binge eating was associated with genetic risk.

### Genotyping

In total, 9,912 participants were genotyped using the Illumina HumanHap550 quad genome-wide SNP genotyping platform by the Wellcome Trust Sanger Institute, Cambridge, UK, and the Laboratory Corporation of America, Burlington, NC. Individuals were excluded from analyses on the basis of excessive or minimal heterozygosity, gender mismatch, individual missingness (>3%), cryptic relatedness as measured by identity by descent (genome-wide IBD >10%) and sample duplication. Individuals were assessed for population stratification using multi-dimensional scaling modeling seeded with HapMap Phase II release 22 reference populations. Individuals of non-European ancestry were removed from further analyses. SNPs with a final call rate of <95%, minor allele frequency (MAF) <1% and evidence of departure from Hardy-Weinberg equilibrium (HWE) (*P* < 5 × 10^−7^) were also excluded from analyses 22. Individuals were imputed to HapMap Phase II (Build 36, release 22) using the Markov Chain Haplotyping software (MACH v.1.0.16) 23.

From these genome-wide data the 32 SNPs previously identified in a large GWAS meta-analysis 19 as being robustly and independently associated with BMI were selected. We also generated weighted polygenic allelic scores using these 32 SNPs. The estimated dosage of each effect allele was weighted by its regression coefficient from Speliotes et al 19. We calculated allelic scores using all 32 SNPs and using 31 SNPs excluding the *FTO* variant.

### Covariates

BMI, weight (kg)/height (m)^2^, was obtained from measured weight and height during a face to face assessment at mean age 13.5 and mean age 15.5 years. BMI at 13.5 was used as the main covariate, if this was missing, BMI at mean age 15.5 was used (correlations between BMI at the two time-points was very high *r* = 0.98). Data on BMI were available on 4,048 adolescents (82.3% of those with available data on binge eating and genotype).

Data on binge eating and genotype were available on 4,360 adolescents (2,406 girls and 1,954 boys) at 14 years, and 3,663 (2,151 girls and 1,512 boys) at 16 years. Overall 4916 adolescents had genotype data and information on binge eating at either age 14 or 16 (2725 girls and 2191 boys).

### Data analyses

We tested the effect of BMI associated SNPs in unadjusted and adjusted logistic regression models where binge eating was the outcome. Adjusted models included age, BMI and gender as covariates. Unadjusted logistic regression models were also conducted stratifying by gender to investigate whether the pattern of association differed between males and females. In order to increase the power of our analyses, we examined the relationship between a weighted allelic risk score and binge eating at either age in an unadjusted logistic regression model. We used effect size coefficients as reported in Speliotes et al. 19 to weight individual SNPs. In order to assess the contribution of the *FTO* variant to the allelic score, two models were tested, one including all 32 SNPs and one comprising 31 SNPs and excluding the *FTO* variant.

Finally, we investigated the relationship between BMI associated SNPs and an ordinal indicator of binge eating (frequency-based) in age stratified adjusted (for gender and BMI) ordinal logistic regression models.

Analyses were run using STATA 12 and the R software package 2.15.2.

## Results

The prevalence of binge eating in adolescence was 5.6% at age 14 years and 11.2% at age 16 years amongst adolescents included in the study (See Table1). Binge eating was more common in girls at both age 14 (7.4% of girls, 3.5% of boys) and 16 (16.1% of girls and 4.4% of boys).

**Table 1:** Characteristics of the sample under study with complete data (genotype and phenotype)

	Age 14 (*n* = 4,360)		Age 16 (*n* = 3,663)	
	Cases (*n* = 245) (5.6%)	Controls (*n* = 4,115)	Cases (*n* = 412) (11.2%)	Controls (*n* = 3,251)
**Gender, females *N* (%)**	177 (72.5%)	2,229 (54.2%)	346 (84.0%)	1,805 (55.5%)
**Age, mean (SD)**	14.05 (0.21)	14.04 (0.19)	16.66 (0.23)	16.68 (0.24)
**BMI, mean (SD)**	21.7 (3.9)	20.2 (3.33)	22.51 (4.0)	21.21 (3.31)

### Relationship between BMI variants and binge eating at either 14 or 16 years of age

Minimally adjusted analyses (adjusted for gender and age) revealed an association in the hypothesised direction between the A allele of rs1558902 (*FTO)* and increased risk of binge eating [odds ratio (OR) = 1.25 (95% CI: 1.10-1.41), *P* = 5.1 × 10^−4^]. This association showed little attenuation after adjusting for BMI (OR = 1.20 (95% CI: 1.05-1.38), *P* = 8.0 × 10^−3^) (Table2). There was also nominal evidence of a protective association (direction opposite to that expected) between rs10150332 (*NRXN3*) and binge eating in unadjusted (OR = 0.85 (95% CI: 0.72-0.99**)**, *P* = 0.038), but not adjusted analyses [OR = 0.88 (95% CI: 0.74-1.04**)**, *P* = 0.13].

**Table 2:** Association between binge eating in adolescence and genetic variants reliably related to BMI

Chrom	Position	SNP	Putative gene	Effect allele^b^	Other allele	RSQR	Minimally adjusted^a^ model (*N* = 4,916)		Conditional on BMI, age, and sex (*N* = 4,048)	
							OR (95% CI)^c^	*P* value	OR (95% CI)^c^	*P* value
**1**	72585028	rs2815752	*NEGR1*	A	G	0.996	1.06 (0.93-1.20)	0.39	1.02 (0.89-1.18)	0.77
**1**	74764232	rs1514175	*TNNI3K*	A	G	0.998	1.08 (0.95-1.22)	0.25	1.03 (0.90-1.19)	0.64
**1**	96717385	rs1555543	*PTBP2*	C	A	0.996	1.02 (0.89-1.16)	0.78	1.01 (0.88-1.17)	0.87
**1**	176156103	rs543874	*SEC16B*	G	A	0.997	1.02 (0.88-1.19)	0.76	0.94 (0.79-1.11)	0.46
**2**	612827	rs2867125	*TMEM18*	C	T	0.999	1.09 (0.92-1.29)	0.30	1.07 (0.89-1.29)	0.49
**2**	25011512	rs713586	*RBJ/ADCY3/POMC*	C	T	0.999	1.02 (0.90-1.15)	0.77	0.97 (0.85-1.12)	0.69
**2**	59156381	rs887912	*FANCL*	T	C	0.997	0.93 (0.81-1.07)	0.32	0.86 (0.74-1.01)	0.058
**2**	142676401	rs2890652	*LRP1B*	C	T	0.989	1.02 (0.87-1.20)	0.80	0.95 (0.79-1.14)	0.59
**3**	85966840	rs13078807	*CADM2*	G	A	0.997	0.99 (0.85-1.16)	0.91	0.95 (0.80-1.13)	0.58
**3**	187317193	rs9816226	*ETV5*	T	A	0.956	1.03 (0.87-1.21)	0.75	0.98 (0.82-1.18)	0.86
**4**	44877284	rs10938397	*GNPDA2*	G	A	0.988	1.10 (0.97-1.25)	0.13	1.11 (0.97-1.28)	0.14
**4**	103407732	rs13107325	*SLC39A8*	T	C	0.997	1.05 (0.83-1.33)	0.69	1.01 (0.77-1.33)	0.93
**5**	75050998	rs2112347	*FLJ35779/HMGCR*	T	G	0.995	1.14 (1.00-1.29)	0.055	1.15 (1.00-1.34)	0.051
**5**	124360002	rs4836133	*ZNF608*	A	C	0.943	1.08 (0.95-1.22)	0.27	1.11 (0.96-1.28)	0.15
**6**	34410847	rs206936	*HMGA1*	G	A	0.988	1.00 (0.86-1.17)	0.97	1.00 (0.84-1.19)	0.98
**6**	50911009	rs987237	*TFAP2B*	G	A	0.999	1.07 (0.91-1.25)	0.42	1.05 (0.87-1.25)	0.62
**9**	28404339	rs10968576	*LRRN6C*	G	A	0.999	1.02 (0.89-1.16)	0.83	1.01 (0.87-1.17)	0.87
**11**	8561169	rs4929949	*RPL27A*	C	T	0.967	0.96 (0.84-1.09)	0.50	0.98 (0.85-1.13)	0.82
**11**	27682562	rs10767664	*BDNF*	A	T	0.997	1.11 (0.95-1.30)	0.18	1.05 (0.88-1.25)	0.61
**11**	47607569	rs3817334	*MTCH2*	T	C	0.998	1.02 (0.90-1.15)	0.78	0.96 (0.84-1.11)	0.60
**12**	48533735	rs7138803	*FAIM2*	A	G	0.998	0.99 (0.87-1.13)	0.93	0.99 (0.86-1.15)	0.92
**13**	26918180	rs4771122	*MTIF3*	G	A	0.931	0.94 (0.81-1.09)	0.41	0.95 (0.80-1.12)	0.54
**14**	29584863	rs11847697	*PRKD1*	T	C	0.969	0.88 (0.64-1.21)	0.41	0.83 (0.59-1.19)	0.30
**14**	79006717	rs10150332	*NRXN3*	C	T	0.996	**0.85 (0.72**-**0.99)**	**0.038**	0.88 (0.74-1.04)	0.13
**15**	65873892	rs2241423	*MAP2K5*	G	A	0.999	1.00 (0.86-1.16)	0.99	0.97 (0.83-1.15)	0.75
**16**	19841101	rs12444979	*GPRC5B*	C	T	0.998	1.13 (0.94-1.36)	0.20	1.06 (0.87-1.31)	0.55
**16**	28793160	rs7359397	*SH2B1*	T	C	0.999	0.97 (0.85-1.09)	0.59	0.94 (0.82-1.09)	0.42
**16**	**52361075**	**rs1558902**	***FTO***	**A**	**T**	**0.997**	**1.25 (1.10**--**1.41)**	**5.1** × **10^−4^**	**1.20 (1.05**-**1.38)**	**8.0** × **10^−3^**
**18**	55990749	rs571312	*MC4R*	A	C	0.999	1.09 (0.94-1.25)	0.27	0.99 (0.84-1.16)	0.89
**19**	39001372	rs29941	*KCTD15*	G	A	0.999	0.97 (0.85-1.10)	0.61	0.98 (0.85-1.14)	0.81
**19**	50894012	rs2287019	*QPCTL/GIPR*	C	T	0.999	0.99 (0.85-1.16)	0.90	0.93 (0.78-1.10)	0.39
**19**	52260843	rs3810291	*TMEM160*	A	G	0.765	1.03 (0.89-1.20)	0.68	0.94 (0.80-1.11)	0.49

a Adjusted for sex.

b BMI increaser allele.

c One tailed binomial sign test indicated that the direction of allelic association with binge eating did not occur significantly more often in the same direction as the known BMI associations (minimally adjusted model *P* = 0.11; conditional model *P* = 0.69).

Bold font indicates statistically significant results (*P* ≤ 0.05).

Females (OR = 1.30, *P* = 3.3 × 10^−4^) displayed slightly stronger evidence for association at the *FTO* locus than males (OR = 1.23, *P* = 0.51), but the difference was not significant (*P* = 0.26) (Table3).

**Table 3:** Association between binge eating in adolescence and genetic variants reliably related to BMI stratified according to sex

Chrom	Position	SNP	Putative gene	Effect allele^a^	Other allele	RSQR	Females, minimally adjusted model (*N* = 2,725)		Males, minimally adjusted model (*N* = 2,191)	
							OR (95% CI)^b^	*P* value	OR (95% CI)^b^	*P* value
**1**	72585028	rs2815752	*NEGR1*	A	G	0.996	0.99 (0.86-1.15)	0.92	**1.32 (1.00**-**1.73)**	**0.047**
**1**	74764232	rs1514175	*TNNI3K*	A	G	0.998	1.09 (0.94-1.26)	0.24	1.03 (0.79-1.33)	0.83
**1**	96717385	rs1555543	*PTBP2*	C	A	0.996	0.99 (0.86-1.14)	0.85	1.14 (0.87-1.49)	0.35
**1**	176156103	rs543874	*SEC16B*	G	A	0.997	0.96 (0.80-1.14)	0.60	1.27 (0.95-1.71)	0.12
**2**	612827	rs2867125	*TMEM18*	C	T	0.999	1.10 (0.91-1.34)	0.32	1.06 (0.74-1.51)	0.75
**2**	25011512	rs713586	*RBJ/ADCY3/POMC*	C	T	0.999	1.02 (0.89-1.18)	0.75	1.00 (0.77-1.30)	0.98
**2**	59156381	rs887912	*FANCL*	T	C	0.997	0.96 (0.81-1.12)	0.57	0.86 (0.64-1.15)	0.31
**2**	142676401	rs2890652	*LRP1B*	C	T	0.989	1.06 (0.88-1.28)	0.53	0.89 (0.63-1.27)	0.52
**3**	85966840	rs13078807	*CADM2*	G	A	0.997	0.96 (0.81-1.15)	0.69	1.08 (0.79-1.48)	0.63
**3**	187317193	rs9816226	*ETV5*	T	A	0.956	1.00 (0.83-1.21)	0.96	1.11 (0.77-1.60)	0.56
**4**	44877284	rs10938397	*GNPDA2*	G	A	0.988	1.11 (0.96-1.28)	0.17	1.09 (0.83-1.42)	0.53
**4**	103407732	rs13107325	*SLC39A8*	T	C	0.997	1.09 (0.83-1.43)	0.55	0.94 (0.57-1.55)	0.79
**5**	75050998	rs2112347	*FLJ35779/HMGCR*	T	G	0.995	**1.21 (1.04**-**1.40)**	**0.012**	0.92 (0.70-1.21)	0.55
**5**	124360002	rs4836133	*ZNF608*	A	C	0.943	1.07 (0.92-1.23)	0.39	1.11 (0.85 -1.45)	0.45
**6**	34410847	rs206936	*HMGA1*	G	A	0.988	1.01 (0.85-1.21)	0.89	0.97 (0.69-1.35)	0.85
**6**	50911009	rs987237	*TFAP2B*	G	A	0.999	1.01 (0.84-1.21)	0.93	1.28 (0.93-1.76)	0.14
**9**	28404339	rs10968576	*LRRN6C*	G	A	0.999	1.06 (0.91-1.23)	0.48	0.89 (0.67-1.18)	0.40
**11**	8561169	rs4929949	*RPL27A*	C	T	0.967	0.93 (0.80-1.07)	0.33	1.06 (0.81-1.38)	0.69
**11**	27682562	rs10767664	*BDNF*	A	T	0.997	1.04 (0.87-1.25)	0.64	1.39 (0.98-1.97)	0.056
**11**	47607569	rs3817334	*MTCH2*	T	C	0.998	1.01 (0.87-1.16)	0.90	1.05 (0.81-1.36)	0.72
**12**	48533735	rs7138803	*FAIM2*	A	G	0.998	1.00 (0.86-1.15)	0.97	0.98 (0.75-1.29)	0.90
**13**	26918180	rs4771122	*MTIF3*	G	A	0.931	0.91 (0.77-1.09)	0.30	1.03 (0.75-1.42)	0.84
**14**	29584863	rs11847697	*PRKD1*	T	C	0.969	0.93 (0.65-1.33)	0.69	0.72 (0.36-1.44)	0.33
**14**	79006717	rs10150332	*NRXN3*	C	T	0.996	0.89 (0.74-1.06)	0.19	0.70 (0.49-1.01)	0.047
**15**	65873892	rs2241423	*MAP2K5*	G	A	0.999	1.05 (0.88-1.25)	0.59	0.86 (0.64-1.17)	0.34
**16**	19841101	rs12444979	*GPRC5B*	C	T	0.998	1.06 (0.86-1.30)	0.59	1.44 (0.94-2.21)	0.083
**16**	28793160	rs7359397	*SH2B1*	T	C	0.999	0.97 (0.84-1.11)	0.63	0.97 (0.74-1.26)	0.81
**16**	**52361075**	**rs1558902**	***FTO***	**A**	**T**	**0.997**	**1.30 (1.13**-**1.49)**	**3.3** × **10^−4^**	1.23 (0.92-1.64)	0.51
**18**	55990749	rs571312	*MC4R*	A	C	0.999	1.04 (0.88-1.23)	0.62	1.23 (0.92-1.64)	0.17
**19**	39001372	rs29941	*KCTD15*	G	A	0.999	0.96 (0.83-1.12)	0.61	0.98 (0.75--1.29)	0.90
**19**	50894012	rs2287019	*QPCTL/GIPR*	C	T	0.999	1.05 (0.88-1.26)	0.58	0.81 (0.59-1.12)	0.21
**19**	52260843	rs3810291	*TMEM160*	A	G	0.765	1.10 (0.93-1.31)	0.27	0.84 (0.62-1.14)	0.26

a BMI increaser allele.

b One tailed binomial sign test indicated that the direction of allelic association with binge eating did not occur significantly more often in the same direction as the known BMI associations (females *P* = 0.11; males *P* = 0.298).

Bold font indicates statistically significant results (*P* ≤ 0.05).

Amongst girls there was nominal association between rs2112347 (*FLJ35779/HMGCR*) and binge eating in the expected direction (OR=1.21 (95%CI: 1.04-1.40), *P* = 0.012) (see Table3), whilst in boys there was nominal evidence of association between rs2815752 (*NEGR1*) and binge eating in the expected direction (OR = 1.32 (95%CI: 1.00-1.73, *P* = 0.047) and rs10150332 (*NRXN3*) and bingeing in the direction opposite to expected (OR = 0.70 (95%CI: 0.49-1.01), *P* = 0.047).

### Frequency of binge eating

Ordinal regression models confirmed the association between the A allele of rs1558902 (*FTO*) and higher binge eating frequency at age 14 years [OR = 1.21 (95%CI: 1.01-1.46), *P* = 0.04] and at 16 years [OR = 1.28 (95% CI: 1.10-1.48), *P* = 1.0 × 10^−3^]. In other words, the presence of the A allele increased the odds of higher binge eating frequency (from never to less than monthly, monthly, weekly, and more than weekly) by 28% for each increase in level at age 16 (see Table4).

**Table 4:** Associations between frequency of binge eating in adolescence and genetic variants reliably related to BMI

Chrom	Position	SNP	Putative gene	Effect allele^b^	Other allele	Minimally adjusted model^a^, age 14 (*N* = 4,359)		Minimally adjusted model^a^, age 16 (*N* =3,663)	
						OR (95% CI)^b^	*P* value	OR (95% CI)^b^	*P* value
**1**	72585028	rs2815752	*NEGR1*	A	G	0.98 (0.81-1.19)	0.87	1.10 (0.95-1.28)	0.19
**1**	74764232	rs1514175	*TNNI3K*	A	G	1.00 (0.83-1.20)	0.98	**1.17 (1.01**-**1.36)**	**0.03**
**1**	96717385	rs1555543	*PTBP2*	C	A	0.95 (0.79-1.15)	0.62	1.06 (0.92-1.24)	0.38
**1**	176156103	rs543874	*SEC16B*	G	A	0.97 (0.78-1.22)	0.84	1.03 (0.87-1.23)	0.70
**2**	612827	rs2867125	*TMEM18*	C	T	1.12 (0.87-1.44)	0.38	1.14 (0.93-1.39)	0.19
**2**	25011512	rs713586	*RBJ/ADCY3/POMC*	C	T	1.11 (0.92-1.33)	0.26	0.94 (0.81- 1.09)	0.41
**2**	59156381	rs887912	*FANCL*	T	C	0.96 (0.78-1.17)	0.70	0.86 (0.73-1.01)	0.07
**2**	142676401	rs2890652	*LRP1B*	C	T	0.98 (0.77-1.25)	0.90	1.00 (0.83-1.21)	0.97
**3**	85966840	rs13078807	*CADM2*	G	A	1.13 (0.91-1.41)	0.26	0.93 (0.77-1.23)	0.46
**3**	187317193	rs9816226	*ETV5*	T	A	0.98 (0.77-1.25)	0.89	0.94 (0.78-1.15)	0.60
**4**	44877284	rs10938397	*GNPDA2*	G	A	1.18 (0.98-1.42)	0.08	1.04 (0.89-1.20)	0.62
**4**	103407732	rs13107325	*SLC39A8*	T	C	0.97 (0.68-1.39)	0.88	1.11 (0.83-1.48)	0.46
**5**	75050998	rs2112347	*FLJ35779/HMGCR*	T	G	1.11 (0.91-1.35)	0.29	1.09 (0.93-1.27)	0.27
**5**	124360002	rs4836133	*ZNF608*	A	C	1.19 (0.98-1.44)	0.07	1.04 (0.89-1.20)	0.62
**6**	34410847	rs206936	*HMGA1*	G	A	1.16 (0.93-1.45)	0.19	0.99 (0.82-1.19)	0.94
**6**	50911009	rs987237	*TFAP2B*	G	A	1.08 (0.85-1.37)	0.51	0.94 (0.78-1.15)	0.56
**9**	28404339	rs10968576	*LRRN6C*	G	A	0.89 (0.73-1.09)	0.26	1.07 (0.92-1.26)	0.34
**11**	8561169	rs4929949	*RPL27A*	C	T	0.88 (0.73-1.06)	0.17	0.97 (0.84-1.13)	0.74
**11**	27682562	rs10767664	*BDNF*	A	T	1.22 (0.96-1.55)	0.10	1.09 (0.90-1.31)	0.36
**11**	47607569	rs3817334	*MTCH2*	T	C	**1.21 (1.01**-**1.46)**	**0.03**	0.95 (0.82-1.11)	0.55
**12**	48533735	rs7138803	*FAIM2*	A	G	0.93 (0.77-1.12)	0.44	1.08 (0.93-1.26)	0.30
**13**	26918180	rs4771122	*MTIF3*	G	A	1.02 (0.82-1.27)	0.85	0.99 (0.82-1.18)	0.88
**14**	29584863	rs11847697	*PRKD1*	T	C	0.78 (0.48-1.27)	0.32	0.90 (0.63-1.29)	0.59
**14**	79006717	rs10150332	*NRXN3*	C	T	0.86 (0.68-1.09)	0.21	0.92 (0.77-1.11)	0.41
**15**	65873892	rs2241423	*MAP2K5*	G	A	0.94 (0.75-1.16)	0.55	0.96 (0.81-1.15)	0.67
**16**	19841101	rs12444979	*GPRC5B*	C	T	1.20 (0.90-1.59)	0.21	1.07 (0.86-1.33)	0.53
**16**	28793160	rs7359397	*SH2B1*	T	C	1.00 (0.83-1.20)	0.99	0.93 (0.81-1.09)	0.40
**16**	52361075	rs1558902	*FTO*	A	T	**1.21 (1.01**-**1.46)**	**0.04**	**1.28 (1.10**-**1.48)**	**1.0** × **10^−3^**
**18**	55990749	rs571312	*MC4R*	A	C	1.06 (0.86-1.31)	0.58	1.05 (0.88-1.25)	0.57
**19**	39001372	rs29941	*KCTD15*	G	A	0.97 (0.79-1.18)	0.73	0.91 (0.78-1.06)	0.26
**19**	50894012	rs2287019	*QPCTL/GIPR*	C	T	1.10 (0.86-1.39)	0.44	0.97 (0.81-1.16)	0.76
**19**	52260843	rs3810291	*TMEM160*	A	G	0.94 (0.76-1.17)	0.59	1.03 (0.87-1.24)	0.68

a For age and BMI.

b One tailed binomial sign test indicated that the direction of allelic association with binge eating did not occur significantly more often in the same direction as the known BMI associations (age 14 *P* = 0.43; age 16 *P* = 0.43).

Bold font indicates statistically significant results (*P* ≤ 0.05).

Two SNPs showed nominal association with frequency of binge eating at either age 14 [rs3817334 (*MTCH2*): OR = 1.21 (95%CI: 1.01-1.46), *P* = 0.03] or 16 years [rs1514175 (*TNNI3K*): OR=1.17 (95% CI: 1.01-1.36), *P* = 0.04] in the expected direction; but these did not replicate across ages.

### Polygenic risk score

In unadjusted analyses, the weighted polygenic risk score was strongly positively associated with adolescent binge eating at either age 14 or 16 (*P* = 7.9 × 10^−4^). This association weakened dramatically once *FTO* was removed from the score (*P* = 0.08). The same pattern was seen in females with the 32 allelic variant weighted polygenic risk score being strongly positively associated with binge eating (*P* = 8.6 × 10^−4^), but not after *FTO* was removed from the risk score (*P* = 0.08). The risk score was not strongly associated with binge eating in males (all *P* > 0.05).

## Discussion

This is the first study to investigate the association between binge eating in adolescence and 32 SNPs that have been robustly associated with BMI, and to include the effect of a weighted allelic score of 32 SNPs associated with BMI. We investigated this association across genders, and explored the association between 32 SNPs and increasing frequency of binge eating (as a more sensitive measure of binge eating and a severity indicator).

We found a significant positive (risk-conferring) association between the A allele of the *FTO* SNP (rs1558902) and adolescent binge eating, independent of BMI. This association was also found when investigating binge eating as a frequency-based ordinal outcome (engaging in binge eating weekly, monthly, or less than monthly).

Analyses stratified by gender showed some evidence for gender differences suggesting the *FTO* rs1558902 A allele might confer increased risk of binge eating amongst girls compared to boys, although the absence of an effect on boys might also be due to lower power due to a reduced prevalence of binge eating in boys (a formal test for interaction was not significant).

A weighted allelic risk score derived from 32 variants associated with BMI showed a positive association with binge eating in unadjusted analyses, with a seemingly stronger effect in girls. Removing the rs1558902 (*FTO*) variant from the allelic risk score attenuated the association between the risk score and binge eating, suggesting that: (i) the *FTO* variant is the major variant driving the association with binge eating, and (ii) the association with bingeing is unlikely to be mediated by BMI (in which case we would also expect an allelic score of BMI SNPs to show strong association with bingeing).

The association between SNPs at the *FTO* locus and other EDs [anorexia (AN) and bulimia nervosa (BN)] has been examined in the literature and resulted in conflicting findings. In particular a recent study found an association between the obesity predisposing allele of the *FTO* variant rs9939609 and AN 24 but not with BN (once adjusted for BMI). In contrast an earlier study did not find an association between the same SNP and AN in a smaller sample of 225 patients with AN and 1,351 controls 25.

The rs1558902 polymorphism is in high linkage disequilibrium with the rs9939609 *FTO* SNP (*r*^2^ = 0.93 in CEU HapMap 2) and a recent meta-analysis has shown similarly strong associations between each of these SNPs and overweight/obesity in children and adolescents 26. There is reason to believe that *FTO* locus SNPs may be related to binge eating. Both rs1558902 and rs9939609 have previously been associated with eating behavior, such as loss of control in adolescents 16, food choice and higher intake of energy-dense food in children 27,28, food responsiveness 29, and decreased satiety 30. A recent study also showed a positive association between rs1558902 (*FTO)* and the cognitive restraint subscale from the three factor eating questionnaire (a measure of restrained eating) 17. Taken together these findings suggest that polymorphisms at the *FTO* locus might increase the risk for both binge eating and overweight/obesity via higher palatable food and energy intake and decreased satiety; or that binge eating might be a mediator on the pathway between genotype and increased BMI/obesity.

Our findings of: (a) an association between an *FTO* locus SNP and binge eating, that showed little reduction in strength after conditioning on BMI, (b) the lack of association with other BMI-increasing SNPS, and (c) an association between the allelic risk score and binge eating when *FTO* was included that attenuated markedly after excluding *FTO* from the allelic risk score suggests that the effect of *FTO* on binge eating is not mediated by BMI. Therefore our findings suggest either pleiotropic effects of variants in or near the *FTO* locus, or that binge eating might be on the causal pathway between genetic variants and obesity. We have shown in two large population-based cohorts (including the one under study) that BED significantly increases the odds of overweight/obesity even when taking into account baseline weight 3 (Micali et al., submitted).

Recent findings have highlighted differences in prefrontal cortex activation in the processing of food stimuli associated with *FTO* polymorphisms, with lower activity (hence lower inhibitory control of eating) in this brain region post-prandially in subjects carrying the risk allele 31. Evidence also suggests that *FTO* rs9939609 AA carriers have a different brain response to food and ghrelin expression compared to TT carriers 32.

Thus, evidence from behavioral genetics and neuroimaging studies supports our findings that genetic variation at the *FTO* locus might confer risk of binge eating. In our study, the strongest associations between the *FTO* polymorphism and binge eating were most evident in girls; although this might be due to a lower prevalence of binge eating in boys and hence lower power to detect differences, or a true differential gender effect (although the formal test for interaction was negative).

In gender-stratified analyses some polymorphisms were nominally associated with binge eating: (e.g., a polymorphism in *FLJ35779/HMGCR* in girls, and a polymorphism in *NEGR1* with binge eating in boys). A variant at *MTCH2* was associated with frequency of binge eating at age 14 years, and a polymorphism in *TNNI3K* with frequency of binge eating at age 16 years. These findings might reflect type I error given the large number of statistical comparisons performed in this paper, and the rather weak *P* values. Future studies should aim to replicate these findings. FLJ35779/HMGCR has not, to our knowledge, been previously implicated in appetite regulation. In contrast the *TNNI3K* and *MTCH2* obesity risk-conferring SNPs have both been shown to be positively associated with emotional eating and uncontrolled eating 17. Emotional eating and uncontrolled eating are traits strongly associated with binge eating, and confer risk for binge eating.

Amongst the SNPs studied we did not find an association between binge eating and variants within *MC4R* or *RBJ/ADCY3/POMC*, despite evidence from previous studies that mutations in the melanocortin 4 receptor (MC4R) and the Pro Opio Melanocortin (*POMC*) genes might be implicated in appetite control 33,34. Similarly, polymorphisms in the *BDNF* gene have been shown to be associated with ED phenotypes 35 but were not strongly associated with binge eating in this study. This might be due to low power to detect associations in our study, or to the selection of more severe phenotypes in previous studies (mostly reliant on clinical populations).

Strengths of our study include a relatively large sample size in comparison to previous studies on genetic risk for binge eating, an investigation of binge eating as a dichotomous outcome and of as an ordinal outcome (i.e., frequency of binge eating), and a candidate gene approach based on 32 SNPs robustly associated with BMI. We also investigated a possible relationship between a weighted allelic risk score and binge eating. Limitations include the fact that data on binge eating were collected using self-report, however our questions were validated in a population-based sample and showed high reliability and validity 36. Secondly, this sample mostly includes subjects of European ancestry, which might limit generalizability of findings. Thirdly, despite being one of the larger genetic studies of binge eating, our study has low statistical power in some analyses, partially due to the lower prevalence of binge eating amongst boys, which might have resulted in false negatives in the associations reported. Our study has limited power to detect genetic variants associated with binge eating in adolescence. This is because of our limited sample size and the fact that genetic variants that influence complex traits like binge eating are likely to exhibit relatively small effects. For example in the case of rs1558902 (*FTO*) which has a risk allele frequency of 0.4, assuming 586 cases and 4331 controls (a prevalence of 13%), a heterozygote relative risk of 1.1, a multiplicative disease model (on the risk scale), we only have ∼40% power to detect an association at α = 0.05 using an allelic test of association. This calculation illustrates that our study has low to moderate power to detect association at individual variants if the effect size of these variants is small (as is likely to be the case). However, assuming that one or more variants jointly contribute to binge eating, combining the variants into an allelic score should increase our power to detect association. Whilst an allelic score comprised of BMI associated variants did show some association with binge eating, our results suggest that most of this association was due to the *FTO* variant, rather than the other BMI related SNPs. We were unable to carry out a replication of our findings in an independent sample within the current study; therefore our findings need replication. However, it should be noted that Tanofsky-Kraff et al. 16 observed an association between a SNP at the *FTO* locus and loss-of-control eating; thus, our finding could be considered a replication.

## Conclusion

This study suggests a positive association between a polymorphism in the *FTO* gene and adolescent binge eating, particularly in girls. Taken together with previous research, it appears that variants within or near the *FTO* locus are associated with a preference for energy-dense foods, greater food intake, less sensitivity to satiety cues, and loss-of-control eating episodes, all of which characterize binge eating. A recent GWAS of 339,224 individuals 37 has identified 97 loci associated with BMI; future studies should therefore test the effect of a weighted allelic score incorporating all 97 loci on binge eating. In the absence of a GWAS of binge eating or BED, future larger studies should also aim to confirm our findings and examine potential risk mechanisms or shared pathways between obesity and binge eating. A GWAS of binge eating or BED is likely to be achievable by pooling available samples across the world.
